# Examination of the Effect of Somatotype Profiles on Athletic Performance Indicators in Children Aged 48-72 Months

**DOI:** 10.7759/cureus.45430

**Published:** 2023-09-17

**Authors:** Rukiye Ciftci, Ahmet Kurtoglu

**Affiliations:** 1 Department of Anatomy, Faculty of Medicine, Gaziantep İslam Bilim ve Teknoloji Üniversitesi, Gaziantep, TUR; 2 Faculty of Sports Sciences, Department of Coaching, Bandırma Onyedi Eylül University, Balıkesir, TUR

**Keywords:** agility test, 20-meter sprint test, push-up test, crunches test, somatotype

## Abstract

Introduction: Physical fitness and anthropometric variables are crucial in achieving success in the field of sports. These variables serve as the foundation and platform for children to showcase their athletic abilities. The aim of this study is to examine the impact of somatotype profiles of children aged 48-72 months on athletic performance in order to contribute to talent selection.

Methods: A total of 124 students (62 females, 62 males), aged between 48 and 72 months (mean age of females: 5.75±1.00, mean age of males: 5.68±1.15), participated in the study. Somatotype analysis was performed using the Heath-Carter method. Performance measurements of students included a 20-meter sprint test, flexibility, leg strength, push-up tests, crunches, vertical jump, standing long jump, hand strength, back strength, and hamstring length determination tests.

Results: In this study, there was a significant difference in favor of mesomorphic endomorph in crunches (F=3.914, p=0.013) and push-up (F=4.864, p=0.004) exercises for female children compared to all somatotypes. In male children, although the central group was dominant in athletic performance measurements, there was no statistically significant difference (p>0.05).

Conclusion: Somatotype is a suitable method for enhancing athletic performance and directing individuals to the appropriate sports discipline. Somatotype profiles are not fully developed in children aged 48-72 months. In the later years, children with suitable somatotypes are expected to demonstrate improved athletic performance.

## Introduction

After birth, human beings go through three interconnected processes, especially in the first 20 years of life. These processes are referred to as growth, maturation, and development [1]. As children grow, there are increases in their height, body weight, and organ sizes. Changes in height and body weight are considered the most easily observable indicators of growth [2].

The growth process involves individual-specific differences. Variations in the timing and rate of growth are explained by the concept of physical aptitude [3]. Within the same chronological age, different levels of growth can be observed. Even among children of the same chronological age, those with a higher level of biological development tend to have grown more than their peers. Similarly, children of the same chronological age but with a lower level of biological development tend to grow later or at a slower pace than other children [4].

Different levels of growth within the same chronological age are closely related to biometric and athletic performance as well. Body composition (BC) and performance differences related to growth vary depending on the physical condition of the children [5]. This is because physically active children tend to have advantages in biometric characteristics such as strength, speed, and endurance compared to other children [1]. Knowing he BC is considered the gold standard for predicting children's physical characteristics [6].

BC, along with other environmental factors, is essential to understand the effects of diet, physical exercise, disease, and physical growth on the human body. In fact, the absolute and relative components of body mass change during growth, making them the primary focus of BC studies. Therefore, when selecting assessment methods for children, it is important to be careful. One important way to determine BC is through somatotype analysis.

Somatotype offers a method to assess and classify the general body shape based on three components: endomorphy (relative fatness), mesomorphy (relative muscularity and skeletal development), and ectomorphy (thin, very low-fat content) [7]. While imaging techniques that directly measure body tissues exist, they can be expensive, not universally applicable, and may not assess body shape. Therefore, somatotype can provide an inexpensive method to indirectly inform about BC [8].

The relationships between somatotype and physical performance have also attracted scientific interest in the general population. However, when compared to the extensive literature on children aged 48-72 months, studies are rare. Research conducted on physically active male children found that mesomorphy was associated with higher muscle strength and ectomorphy with lower muscle strength. Ectomorphy and mesomorphy have also been associated with better gains during aerobic fitness training in children [9].

The relationship between somatotype and physical fitness in children has significant implications for public health because good cardiorespiratory fitness in childhood is associated with better metabolic risk factors, and this relationship persists into adulthood [10].

When evaluating performance tests used for talent selection, neglecting the influence of training history and/or biological development can result in the selection of individuals who are more developed and/or better trained as "talented" rather than those with true talent [11]. The aim of this study is to contribute to talent selection by determining the somatotype profiles of children aged 48-72 months and examining their impact on athletic performance.

## Materials and methods

A total of 124 students (62 females, 62 males), aged between 48 and 72 months (mean age for females: 5.75±1.00, mean age for males: 5.68±1.15), participated in the study. Sample size calculation was performed using the G-Power 3.1.7 software package, with a 95% confidence interval, α=0.05, and 1-β=0.80 [12]. Each school, parent/guardian, and student were informed, gave consent, and had the right to withdraw from the study. The study included students who a) were in kindergarten, preschool, or first grade; b) were aged between 48 and 72 months; c) had no mental issues; and d) were enrolled in the same school. The study excluded students who a) did not want to participate in the study; b) were not within the desired age range; c) had mental problems; and d) had lost their ability to walk. A "Voluntary Consent Form" was obtained from the families of all participants. Participants were informed about the tests to be conducted. 

This research was conducted in accordance with the principles outlined in the Helsinki Declaration. Ethical approval for the research was obtained from the Bandırma Onyedi Eylül University Institute of Health Sciences Ethics Committee, with approval number 2023/3.

Data collection

The study only included students aged between 48 and 72 months who were enrolled in the same school. After obtaining ethical approval for the study, data collection began. The students' performance measurements included a 20-m sprint test, agility test, crunches and push-up tests, vertical jump, standing long jump, hand strength, back strength, and hamstring length determination tests.

Somatotype measurement

Height was measured using a stadiometer with 0.1 cm sensitivity, and weight was measured using a segmental body composition analysis device (model: BC 418; Tanita Corporation, Tokyo, Japan) with 0.1 kg sensitivity. BMI was calculated using the formula weight (kg)/height (m²). Subsequently, body circumferences (flexed and stretched upper arm circumference and calf circumference) were measured to the nearest 0.1 cm using a flexible but non-stretchable tape (Holtain Ltd., Croswell, UK). The bi-epicondylar humerus and femur widths were measured to the nearest 0.1 cm using a bicondylar caliper (Holtain Ltd., Croswell, UK). Participant skinfold thickness was determined using a skinfold caliper (Holtain Ltd., Croswell, UK) in four regions (triceps, suprailiacus, subscapula, calf). Somatotype calculations were performed using the "Somatotype for Windows 1.2.5 Trial Version" software.

20 m sprint test

A 20-m course was marked with photocells placed at the beginning and end of the track. Participants started the sprint from 50 cm behind the starting line. Two trials were conducted, and the best time was recorded [13].

Crunch test

Participants lay on their backs with their hands clasped behind their heads and knees bent slightly toward the abdomen (knees at a 90-degree angle), with the soles of their feet flat on the ground. They were instructed to raise themselves upward, bringing their elbows forward, and touch their knees at the end of the movement. Throughout the entire movement, they ensured that their hands remained clasped behind their heads. Upon returning to the starting position at the command of "Ready... Begin," they attempted to perform as many repetitions as possible within a 30-s period. The result was recorded, and the test was performed once [14].

Push-up test

Participants assumed the full push-up position with straight and tense elbows. Their body was lowered until it touched the ground, and then they pushed their body back up to the starting position with straight and tense elbows. They performed as many maximal push-ups as possible within 30 s, and the result was recorded [14].

Standing long-jump test

In this test, participants placed the number '0' on a line, at the beginning of a steel meter. They stood with the meter strip in the middle of both feet. Participants were instructed to jump as far as they could. After the jump, the last point where the participants' feet landed was marked and measured. For reliability, participants performed the test twice, and the best distance was recorded [14].

Vertical jump test

The vertical jump performance of the athletes was measured using the electronic Smart Speed Lite system. The vertical jump test was conducted following a 15-minute active warm-up, consisting of 5 minute of running, 5 minute of short sprints, and 5 minutes of stretching and mobility exercises. Participants were instructed to jump to the highest point they could when they felt ready, and then they landed back on the mat. The athletes' jump distances were electronically measured in centimeters, and the best of three attempts was recorded [14].

Back strength

Back strength was measured using a back dynamometer. Participants placed their feet on the dynamometer platform with their knees extended, and with their arms extended, their backs straight, and their bodies slightly leaning forward, they pulled the dynamometer bar that they gripped with their hands vertically upward. Two attempts were made, and the best result was recorded [15].

Handgrip strength

Participants stood with their arms at their sides while holding a hand dynamometer, with the measuring portion of the dynamometer facing outward [16]. They squeezed the dynamometer with maximum force, and the best measurement was taken twice for both hands and recorded in kilograms [17].

Hamstring length (sit-reach test)

The Baseline® device (Cooper Institute/YMCA, AAHPERD, New York, USA) was used for the test. Before the measurement, the subject was asked to place their heels on the test device in a long sitting position and then bend forward three times to warm up. Afterward, the subject's arm length on the device was determined, and they were asked to stretch forward as far as possible by pressing on the measuring device with their fingertips without lifting their knees. The measurements were taken three times, and the average was recorded [18].

Procedure

The study was conducted at a special school. First, somatotype measurements were taken from the participating students, and then the students were prepared for performance evaluations. Students were shown a brief warm-up exercise, and they were asked to run on a 20-m track. After a 10-minute rest, students were asked to perform sit-ups for 30 s, and the number of crunches performed was recorded. After another 10-minute rest, students were asked to do push-ups within 30 s, and the number of push-ups performed was noted. Following a 10-minute rest, students were asked to jump to the farthest point possible on a platform with a starting point and the farthest point jumped was recorded. Students also jumped to the farthest point they could on a mat, and the farthest distance they jumped was recorded electronically. Then, hand strength was measured using a hand dynamometer, and hamstring muscle strength was assessed with the sit-reach test.

Statistical analysis

Statistical analysis of the research was conducted using Statistical Product and Service Solutions (SPSS, ver. 25; IBM, Chicago, USA). Normality analysis of the data was performed using the Kolmogorov-Smirnov test, which determined that the data followed a normal distribution. The homogeneity of variances was assessed using Levene's test. Based on these results, a one-way analysis of variance (ANOVA) test was applied to analyze performance indicators according to somatotype categories (endomorphic mesomorph, mesomorphic endomorph, endomorph-mesomorph, and central). The significance level in the study was set at 0.05.

## Results

The demographic information and anthropometric measurement results related to the participants' body dimensions are presented in Table 1. A total of 150 children were assessed in the study. However, 36 children who did not meet the inclusion criteria were excluded from the study. As a result, a total of 124 children (62 females, 62 males) were included in the study. The mean age of the females was 5.75±1.00, while the mean age of the males was 5.68±1.15. In the study, four somatotype profiles were obtained: mesomorphic endomorph, endomorph-mesomorph, endomorphic mesomorph, and central.

**Table 1:** ANOVA test results of demographic characteristics according to somatotype were given Data shown as mean ± SD, nf: number of women, nm: number of men, BMI: body mass index, p<0.05 indicating a statistically significant difference between the groups.

Parameters	Gender	Mesomorphic endomorph (n_f_=14, n_m_=14 )	Endomorph-mesomorph (n_f_=9, n_m_=9 )	Endomorphic-mesomorph (n_f_=31, n_m_=31 )	Central (n_f_=8, n_m_=8 )	p
Age (year)	Female	6.50±0.85	5.66±1.00	5.58±1.05	6.62±0.51	0.105
	Male	6.38±0.97	5.40±0.84	5.78±1.15	6.00±1.30	0.115
Height (cm)	Female	120.96±9.63	114.77±9.82	116.09±11.05	126.57±6.18	0.053
	Male	125.22±6.89	120.30±8.59	117.96±8.76	123.12±5.59	0.122
Weight (kg)	Female	23.69±4.80	21.16±4.28	21.18±4.67	25.61±6.52	0.103
	Male	28.02±7.42	22.22±3.70	23.17±5.31	23.70±2.98	0.118
BMI (kg/m^2^)	Female	16.50±3.03	15.52±1.13	15.47±1.16	16.85±1.16	0.111
	Male	15.34±1.25	16.73±1.91	16.69±2.44	16.20±1.57	0.137
Leg circumference (cm)	Female	24.62±2.44	27.68±9.74	22.98±4.44	25.04±3.31	0.115
	Male	26.26±2.57	24.16±1.75	24.62±2.45	25.37±1.53	0.064
Arm Circumference (cm)	Female	18.13±1.43	17.52±1.06	17.16±1.25	18.17±3.19	0.201
	Male	18.80±2.38	17.05±1.21	18.12±1.81	17.66±1.15	0.114
Bi-Femoral Breadth (cm)	Female	7.34±0.71	7.18±0.71	6.99±0.68	7.41±0.71	0.315
	Male	7.25±0.79	6.65±0.63	7.03±0.69	7.08±0.47	0.193
Bi-Humeral Breadth (cm)	Female	5.20±0.29	5.06±0.29	5.00±0.13	5.28±0.38	0.011*
	Male	5.17±0.25	4.91±0.33	5.12±0.31	5.05±0.10	0.122
Triceps Skinfold (mm)	Female	9.40±3.51	10.57±3.65	9.80±2.72	10.65±3.76	0.760
	Male	13.31±3.42	9.06±2.43	11.53±3.28	10.17±1.24	0.006*
Subscapular Skinfold (mm)	Female	7.39±1.83	7.20±2.25	7.68±1.90	8.77±4.43	0.554
	Male	10.28±5.56	7.13±1.62	8.88±3.04	7.40±1.32	0.124
Suprailiac Skinfold (mm)	Female	8.59±4.61	6.94±1.22	8.79±3.19	8.25±5.14	0.613
	Male	13.12±6.18	8.82±2.62	9.74±3.47	8.50±3.27	0.018*
Calf Skinfold (mm)	Female	13.77±4.46	12.71±1.99	12.69±3.18	14.28±4.34	0.614
	Male	17.90±6.51	13.77±4.50	15.05±5.89	12.37±2.36	0.107

In Table 1, there was a significant difference in bi-humeral breadth measurement among female children for all somatotype profiles, while among male children, there was a statistically significant difference in triceps skinfold and suprailiac skinfold values (p<0.05) (Table 1).

**Figure 1:**
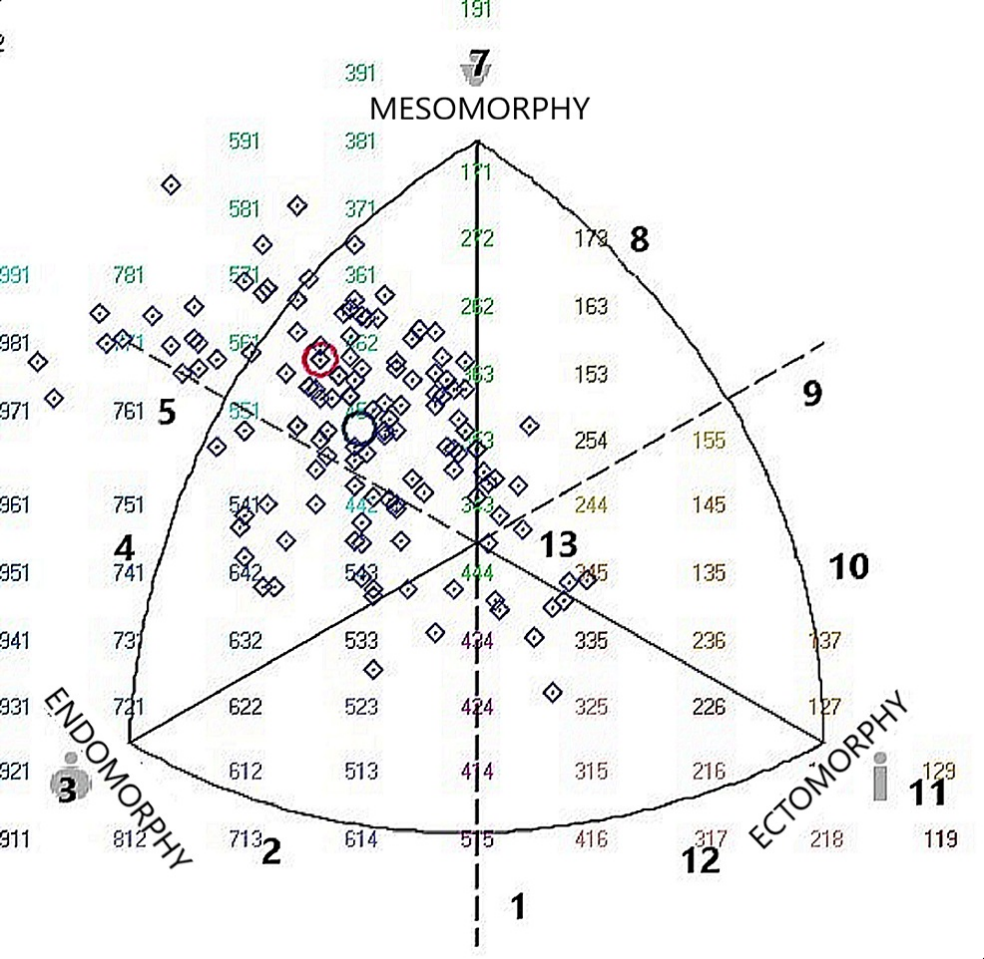
Somatoplot representations of the somatotype characteristics of our study 1: endomorph ectomorph, 2: ectomorphic endomorph, 3: balanced endomorph, 4: mesomorphic endomorph, 5: mesomorph endomorph, 6: endomorphic mesomorph, 7: balanced mesomorph, 8: ectomorphic mesomorph, 9: mesomorph ectomorph, 10: mesomorphic ectomorph, 11: balanced ectomorph, 12: endomorphic ectomorph, 13: central. It is the author's original work.

In Table 2, ANOVA results of performance parameters of female participants according to somatotype were analyzed. Accordingly, there was a statistically significant difference between push-up test results (F3-58=4.864, p=0.004) and crunches test results (F3-58=3.914, p=0.013) of female participants (Table 2, Figure 1).

**Table 2:** Comparison of athletic performance of female participants according to somatotypes according to ANOVA test results Data shown as mean ± SD, nf: number of women, nm: number of men, p<0.05 indicating a statistically significant difference between the groups.

Parameters	Somatotype	Mean±S.D.	F_(3-58)_	p
Back Strenght (kg)	Mesomorphic Endomorph (n=14)	23.85±9.60	0.218	0.884
	Endomorph-Mesomorph (n=9)	16.66±13.32		
	Endomorphic Mesomorph (n=31)	17.03±13.17		
	Central (n=8)	20.62±13.47		
Leg Strenght (kg)	Mesomorphic Endomorph (n=14)	23.76±6.97	1.694	0.183
	Endomorph-Mesomorph (n=9)	32.58±8.58		
	Endomorphic Mesomorph (n=31)	28.85±9.81		
	Central (n=8)	27.95±6.22		
20 m Sprint (sec)	Mesomorphic Endomorph (n=14)	5.36±1.30	1.127	0.346
	Endomorph-Mesomorph (n=9)	5.53±0.98		
	Endomorphic Mesomorph (n=31)	5.67±1.80		
	Central (n=8)	4.90±0.22		
Flexibility (cm)	Mesomorphic Endomorph (n=14)	10.57±6.84	0.786	0.507
	Endomorph-Mesomorph (n=9)	15.22±1.20		
	Endomorphic Mesomorph (n=31)	13.61±5.04		
	Central (n=8)	14.75±2.13		
Push-up	Mesomorphic Endomorph (n=14)	13.21±9.51	4.864	0.004*
	Endomorph-Mesomorph (n=9)	5.33±6.57		
	Endomorphic Mesomorph (n=31)	5.64±4.96		
	Central (n=8)	8.66±4.22		
Crunches	Mesomorphic Endomorph (n=14)	15.21±5.96	3.914	0.013*
	Endomorph-Mesomorph (n=9)	10.11±5.62		
	Endomorphic Mesomorph (n=31)	9.45±7.10		
	Central (n=8)	16.00±1.67		
Standing Long Jump (cm)	Mesomorphic Endomorph (n=14)	85.78±20.78	0.806	0.494
	Endomorph-Mesomorph (n=9)	80.44±25.23		
	Endomorphic Mesomorph (n=31)	75.22±28.70		
	Central (n=8)	88.83±16.66		
Vertical Jump (cm)	Mesomorphic Endomorph (n=14)	14.00±4.94	0.294	0.830
	Endomorph-Mesomorph (n=9)	12.44±5.36		
	Endomorphic Mesomorph (n=31)	12.60±4.99		
	Central (n=8)	13.16±4.02		
Handgrip Strenght-L (kg)	Mesomorphic Endomorph (n=14)	9.88±2.60	2.098	0.111
	Endomorph-Mesomorph (n=9)	8.68±2.91		
	Endomorphic Mesomorph (n=31)	8.02±2.80		
	Central (n=8)	10.16±2.01		
Handgrip Strenght-R (kg)	Mesomorphic Endomorph (n=14)	9.05±2.31	1.466	0.234
	Endomorph-Mesomorph (n=9)	8.75±3.86		
	Endomorphic Mesomorph (n=31)	8.10±3.03		
	Central (n=8)	10.75±1.97		

In Table 3, ANOVA test results between male participants' somatotype results and performance parameters were analyzed. Accordingly, it was concluded that the performance indicators in men were not affected by the somatotype (p>0.05) (Table 3, Figure 1).

**Table 3:** Comparison of athletic performance of male participants according to somatotypes according to ANOVA test results Data shown as mean ± SD, nf: number of women, nm: number of men, p<0.05 indicating a statistically significant difference between the groups.

Parameters	Somatotype	Mean±S.D.	F_(3-59)_	p
Back Strenght (kg)	Mesomorphic Endomorph (n=14)	24.00±4.24	0.720	0.544
	Endomorph-Mesomorph (n=9)	22.00±8.83		
	Endomorphic Mesomorph (n=31)	23.31±5.01		
	Central (n=8)	26.20±9.11		
Leg Strenght (kg)	Mesomorphic Endomorph (n=14)	23.94±6.22	1.188	0.322
	Endomorph-Mesomorph (n=9)	27.35±7.77		
	Endomorphic Mesomorph (n=31)	27.16±7.81		
	Central (n=8)	29.25±8.09		
20 m Sprint (sec)	Mesomorphic Endomorph (n=14)	5.80±0.68	1.899	0.139
	Endomorph-Mesomorph (n=9)	5.42±0.69		
	Endomorphic Mesomorph (n=31)	5.59±0.86		
	Central (n=8)	5.07±0.45		
Flexibility (cm)	Mesomorphic Endomorph (n=14)	16.33±4.96	0.512	0.676
	Endomorph-Mesomorph (n=9)	17.35±4.60		
	Endomorphic Mesomorph (n=31)	15.37±4.86		
	Central (n=8)	16.0±1.66		
Push-Up	Mesomorphic Endomorph (n=14)	5.83±4.54	0.430	0.732
	Endomorph-Mesomorph (n=9)	5.50±4.27		
	Endomorphic Mesomorph (n=31)	6.93±5.86		
	Central (n=8)	7.75±3.91		
Crunches	Mesomorphic Endomorph (n=14)	11.88±5.68	0.232	0.874
	Endomorph-Mesomorph (n=9)	13.40±7.96		
	Endomorphic Mesomorph (n=31)	11.58±6.51		
	Central (n=8)	12.50±2.92		
Standing Long Jump (cm)	Mesomorphic Endomorph (n=14)	81.16±18.67	1.305	0.281
	Endomorph-Mesomorph (n=9)	89.40±22.67		
	Endomorphic Mesomorph (n=31)	81.53±24.42		
	Central (n=8)	96.68±17.62		
Vertical Jump (cm)	Mesomorphic Endomorph (n=14)	12.33±2.58	1.914	0.127
	Endomorph-Mesomorph (n=9)	12.70±5.55		
	Endomorphic Mesomorph (n=31)	14.90±4.90		
	Central (n=8)	15.50±3.02		
Handgrip Strenght-L (kg)	Mesomorphic Endomorph (n=14)	8.91±2.06	1.463	0.233
	Endomorph-Mesomorph (n=9)	9.62±2.90		
	Endomorphic Mesomorph (n=31)	8.07±2.70		
	Central (n=8)	9.72±3.06		
Handgrip Strenght-R (kg)	Mesomorphic Endomorph (n=14)	9.46±2.27	1.758	0.164
	Endomorph-Mesomorph (n=9)	8.35±1.67		
	Endomorphic Mesomorph (n=31)	8.31±2.78		
	Central (n=8)	10.26±2.65		

## Discussion

In this study, where we aimed to determine the somatotype profiles of children aged 48-72 months and examine their impact on athletic performance to contribute to talent selection, we found a significant difference in favor of mesomorphic endomorph in crunches and push-up exercises among female children for all somatotype profiles. However, among male children, although the central group was dominant in athletic performance measurements, there was no statistically significant difference.

Somatotype is determined by anthropometric measurements and describes an individual's morphological structure. The somatotype profile is essential in determining an individual's suitability for a particular sport. Physical fitness tests have been used as criteria for examining the relationship between somatotype and athletic performance. Strength, endurance, and speed tests, especially flexibility, balance, hand-eye coordination, or limb movement speed tests, are more related to somatotype than extreme ends of physical performance. In particular, somatotype plays an important role in exercises such as sit-ups, push-ups, vertical jumping, and standing long-jump tests [19,20]. In our study, when we compared the athletic performance of male and female children to all somatotype profiles, although differences were observed in male children, the results were not statistically significant.

Especially in children, poor nutrition and sedentary behavior, particularly in the technological age, can increase body fat percentage and disrupt body composition. It has been found that individuals with an endomorphic somatotype have a higher body fat percentage compared to other somatotype profiles [21]. Widiyani et al. [21] found in their study that Nigerian primary school girls were more endomorphic. In our study, the majority of female children had mesomorphic endomorph and central somatotype profiles. Monyeki et al. mentioned that preschool girls tend to be dominant in mesomorphy throughout age groups but less dominant in endomorphy [22]. Ayan et al. [23] reported in their study that girls had somatotype values of endomorph-mesomorph, and Jurak et al. [24] reported mesomorphic endomorph. Although the majority of the girls and boys included in our study had an endomorphic mesomorph somatotype profile, all athletic performance values were in the mesomorphic endomorph and central groups.

When examining the relationships between somatotype structure and performance skills, it was observed that the majority of girls had a mesomorphic endomorph somatotype, and they performed more push-ups compared to boys. In girls, the central somatotype was found to be more effective in push-up and crunch performances, which are important indicators of strength.

Revan et al. examined the performance parameters such as the vertical jump and standing long jump according to somatotype in taekwondo athletes. They reported that foreign male taekwondo athletes had a more mesomorphic-ectomorphic somatotype, while Turkish male taekwondo athletes had a somatotype of mesomorph-ectomorph [25]. Considering that these individuals are actively involved in sports, we believe that the main reason for the different results in our study is that the sample in our study was not actively involved in any sports branch.

The population of our study consisted of preschool and first-grade students, with mesomorphic endomorph being the dominant somatotype in most performance measures for girls and central for boys. The children in our study need to have the appropriate somatotype for the sports they will choose in the future.

Determining somatotypes in children aged 48-72 months is important because, at this age, children's somatotypes are not fully developed. However, according to the information obtained from the literature, planning should be made for the ectomorphic body type to increase athletic performance [26].

Limitations

In this study, we examined athletic performance values according to somatotypes in children aged 48-72 months. If older children were included in the study, we could have made interpretations about how athletic performance changes with age. In future studies, conducting the same study in the same sample or in a different sample with older children could provide us with clearer information on which sports children should be directed to.

## Conclusions

Strength, speed, endurance, agility, and flexibility parameters are common and fundamental motor skills present in most sports. The ability to perfect these skills, both generally and sport-specific, contributes to providing athletes with physical fitness and enhancing sport-specific performance. When reviewing the literature, it is observed that elite-level athletes tend to have ectomorphic or ectomorphic mesomorphic body types. Therefore, in choosing an appropriate sports branch for preschoolers or determining the required body type for achieving the target performance in existing athletes, planning for an ectomorphic body type should be considered. It is expected that the results of our research will contribute to relevant scientists, physical education teachers, coaches, experts involved in talent screening projects, children, and parents.
